# GPR35 in Intestinal Diseases: From Risk Gene to Function

**DOI:** 10.3389/fimmu.2021.717392

**Published:** 2021-11-01

**Authors:** Berna Kaya, Hassan Melhem, Jan Hendrik Niess

**Affiliations:** ^1^ Department of Biomedicine, University of Basel, Basel, Switzerland; ^2^ Department of Gastroenterology/Hepatology, Clarunis - University Center for Gastrointestinal and Liver Diseases, Basel, Switzerland

**Keywords:** GPR35, microbiota, metabolites, ligand-receptor interactions, risk variants, inflammatory bowel diseases

## Abstract

Diet and gut microbial metabolites mediate host immune responses and are central to the maintenance of intestinal health. The metabolite-sensing G-protein coupled receptors (GPCRs) bind metabolites and trigger signals that are important for the host cell function, survival, proliferation and expansion. On the contrary, inadequate signaling of these metabolite-sensing GPCRs most likely participate to the development of diseases including inflammatory bowel diseases (IBD). In the intestine, metabolite-sensing GPCRs are highly expressed by epithelial cells and by specific subsets of immune cells. Such receptors provide an important link between immune system, gut microbiota and metabolic system. Member of these receptors, GPR35, a class A rhodopsin-like GPCR, has been shown to be activated by the metabolites tryptophan-derived kynurenic acid (KYNA), the chemokine CXCL17 and phospholipid derivate lysophosphatidic acid (LPA) species. There have been studies on GPR35 in the context of intestinal diseases since its identification as a risk gene for IBD. In this review, we discuss the pharmacology of GPR35 including its proposed endogenous and synthetic ligands as well as its antagonists. We elaborate on the risk variants of GPR35 implicated in gut-related diseases and the mechanisms by which GPR35 contribute to intestinal homeostasis.

## Introduction

The gastrointestinal tract is home to the vast majority of microbiota in the body most of which are colonized in the distal intestine (1). The gut microbiota consists of microorganisms including bacteria, viruses, parasites, and fungi. These microorganisms co-evolved with the host to form a symbiotic relationship. Intestinal microbiota is crucial for the regulation of the host metabolism and immunity. In turn, the intestinal immune system is tasked to establish tolerance towards the microbial community and to prevent pathogen invasion. The intestine is covered by an epithelial cell layer which forms a physical and biochemical barrier between the microbiota and the host (2). A compromised barrier integrity and/or dysregulated immune responses causes loss of tolerance to microbials and/or food antigens. This loss of tolerance is central to the pathogenesis of chronic intestinal disorders such as celiac disease, food allergies, and inflammatory bowel diseases (IBD) including Crohn’s disease (CD) and ulcerative colitis (UC) in genetically susceptible individuals (3).

Since the incidence of IBD patients has rapidly increased in developing countries, it is unlikely that genetic factors alone are the main cause of rising rates of IBD (4). Thus, doubtlessly, environmental exposure significantly contributes to the development of IBD. Among environmental factors, dietary nutrients are increasingly recognized for their contribution to the pathogenesis of IBD (5, 6). Specifically, a fiber-rich diet lowers risk of Crohn’s disease whereas a “western” diet containing poly-unsaturated fatty acids, and trans-fats is associated with IBD (7, 8). In this context, there is increasing evidence that host and/or microbiota-derived metabolites shape the host intestinal immune system by activating signaling cascades in epithelial and immune cells. These metabolites can evoke an immunological response through distinct pathways. Certain metabolites such butyrate and propionate can act as histone deacetylase inhibitors or transcriptional coactivators and thereby control gene expression intracellularly. In addition, dietary metabolites can signal *via* G protein-coupled receptors (GPCRs) (9). GPCRs can recognize host-derived metabolites such as medium- and long-chain fatty acids or secondary metabolites that undergo fermentation by the gut microbiota including fiber-derived short-chain fatty acids. Short-chain fatty acids such as butyrate, acetate, propionate, and nicotinic acid activate anti-inflammatory responses and bind to GPCRs including GPR41, GPR43 and GPR109A on epithelial and immune cells such as dendritic cells and regulatory T cells (10–12). Beside influencing host immunity and mucosal barrier integrity, dietary metabolites may affect the composition of the intestinal microbiota. This review focuses on the metabolites-sensing GPR35, which is a newly characterized receptor that profoundly affects the functions of immune and nonimmune cells in the intestine. we summarize and discuss the experimental findings emphasizing the importance of GPR35 in intestinal health and disease.

## G Protein-Coupled Receptor (GPR) 35

GPR35 is a rhodopsin-like, 7-transmembrane class A GPCR that was discovered in 1998 (13). The *GPR35* is located on chromosome 2q37.3 in humans that can be alternatively spliced into variants resulting in GPR35a or GPR35b. GPR35a encodes for 309 amino acids whereas GPR35b results in a longer N-terminal domain by 31 amino acids (14). Although GPR35b is rather associated with carcinogenesis due to its expression in gastric and colon cancer cells, GPR35a and GPR35b are localized at similar cellular compartments as well as have similar pharmacology. Hence, the functional differences between the two isoforms are not clear and yet to be studied (14–17). There are orthologues of GPR35 in mouse and rat that share 73 and 72% homology with human GPR35a (hGPR35a) and, in less extensively studied non-mammalian species such as *Xenopus tropicalis* (amphibian) with 33% similarity to the hGPCR35a (18, 19). Recently, *Danio rerio* (zebrafish) homolog of hGPR35 has been cloned which led to identification of two paralogs, *gpr35a* and *gpr35b*, that show ~26% and 24% sequence similarity to human *GPR35* (20).

GPR35 is closely related to cannabinoid receptor GPR55, the nicotinic acid receptor HM74 and LPA receptors LPAR4, LPAR5 and LPAR6 (21, 22). However, GPR35 remains an orphan receptor to date, although several potential ligand candidates have been proposed (reviewed extensively below). In this context, kynurenic acid, a tryptophan metabolite, has been suggested as a putative ligand candidate for GPR35 (23). In addition to kynurenic acid, phospholipid derivates LPA species were explored for their potency to activate GPR35 due to the structural similarity of GPR35 to the LPA receptors (24). Furthermore, CXCL17, a mucosal chemokine, was shown activate GPR35 which led to the suggestion to rename GPR35 as chemokine (C-X-C motif) receptor 8 (CXCR8) (25). In the study where GPR35 was first reported, O’Dowd et al. have shown that the expression of the newly identified GPR35 is abundant in the small intestine in rats (13). Other studies reported similar expression pattern in the small intestine and colon of mice and humans. These studies also indicated significant GPR35 expression in the spleen as well as immune cells including monocytes, macrophages, and dendritic cells. On the other hand, lower levels of GPR35 were evident in the stomach, liver, and kidney. Furthermore, *gpr35a* expression was restricted to the zebrafish intestine at larvae stage (20).

In addition to the expression pattern of GPR35, the polymorphisms in the human gene has been investigated by genome wide association studies (26). Importantly, two SNP variants of GPR35 have been associated with IBD that led to the definition of GPR35 as an IBD risk gene (27, 28). Subsequently, this prompted researchers to understand the functions of GPR35 in the intestine under different physiological conditions.

## Pharmacology of GPR35

### Potential Endogenous Ligands

#### Kynurenic Acid

To understand the function of GPR35, it is crucial to delineate upstream and downstream signaling events of its activation. To this end, Wang and colleagues tested kynurenic acid’s ability to activate GPR35 signaling (23). Kynurenic acid is a tryptophan-derived metabolite that is mostly recognized for its roles in the central nervous system as well as anti-inflammatory properties (23, 29, 30). Of note, higher levels of kynurenic acid is evident in serum of inflammatory bowel disease patients (31). In micromolar concentrations, kynurenic acid induces Ca^2+^ influx in the presence of chimeric G proteins *via* human, mouse and rat GPR35 in Chinese hamster ovary cells (23). Furthermore, kynurenic acid led to GPR35 internalization in HeLa cells. Other studies demonstrated that GPR35 deficiency hinders the kynurenic acid-induced adhesion of human peripheral monocytes as well as ant-inflammatory gene expression in adipocytes in mice (32, 33). Nonetheless, although the potency of kynurenic acid for activating the GPR35 is relatively high in mice and rats, the concentrations of kynurenic acid required are 40- to 100- fold higher in humans (32). In addition, in other studies, kynurenic acid failed to activate human GPR35 even at high concentrations (20, 24). This species selectivity of kynurenic acid/GPR35 axis has led to the discussion whether the interaction is physiological, particularly in humans, which causes kynurenic acid to retain a potential endogenous ligand for GPR35 (34).

#### Lysophosphatidic Acid

GPR35 has also been explored as a receptor for LPA due to its homology to other LPA receptors. Oka et al. have shown that LPA led to Ca^2+^ response, activation of RhoA and ERK phosphorylation in GPR35-transfected cells as well as internalization of GPR35 (24). LPA also induced a transient RhoA activation in vector-transfected control cells which was attributed to the other LPA receptors. LPA is a bioactive phospholipid derivate that is present in cell membrane but also can be produced extracellularly and activate six known GPCRs, LPAR1-6 (35). In a more recent study by Schneditz and colleagues, GPR35 deficiency prevented the LPA-induced Ca^2+^ signaling in bone marrow-derived macrophages (BMDMs). However, the authors hypothesized that the GPR35 deficiency might impair LPA signaling *via* the other LPA receptors (36). GPR35 had not been confirmed as a potential LPA receptor until our group have tested LPA in cells transfected with human GPR35 naturally coupled to an inhibitory G-protein (Gi). In this ligand identification assay, LPA lowered the cAMP signaling suggesting that LPA might induce Gi-mediated signaling (20). The ligand-GPCR interaction studies are challenged by interspecies variances especially including that of GPR35. Despite this complexity, LPA/GPR35 axis have been evident across the species, mouse and zebrafish, where LPA led to GPR35-dependent migration of macrophages as well as TNF cytokine induction marked with activation of NF-kB and ERK pathways. However, it is still not known whether GPR35 directly binds to LPA or regulates LPA signaling by interacting with and modulating the activity of its receptors.

#### CXCL17

CXCL17, whose expression is associated with mucosal sites, has also been proposed as an endogenous agonist of GPR35 (25, 37, 38). Maravillas-Montero et al. determined that GPR35-transfected cells migrate towards CXCL17 (25, 37, 38). Since CXCL17 acted on GPR35 in nanomolar concentrations that are in a physiological range as opposed to kynurenic acid, the authors suggested to change the nomenclature of GPR35 into CXCR8. However, subsequent studies reported that CXCL17 failed to induce migratory or signaling responses in GPR35-expressing cells (39, 40). In addition, kynurenic acid and zaprinast reduced the effects of CXCL17 in neuropathic pain model in mice further supporting the existence of CXCL17 receptor(s) other than GPR35 (41).

In addition to kynurenic acid, LPA and CXCL17, there have been studies describing potency of other endogenously available molecules for GPR35 including 5,6-dihydroxyindole-2-carboxylic acid (DHICA), 3,3,5-triiodothyronine (reverse T3), and guanosine 3',5'-cyclic monophosphate (cGMP) (**Table 1**) (44, 46). However, these have not been pursued experimentally as GPR35 ligand candidates following their initial identification. In addition to attempts in deorphanizing the GPR35, it is important to mention that Schneditz et al. reported a ligand-independent basal activity of GPR35 *via* its interaction with the Na/K-ATPase (36).

**Table 1:** Proposed agonists and antagonists of GPR35.

Potential Endogenous Ligands	Source in the intestine	EC50 (human)	EC50 (mouse)	EC50 (rat)	References
Kynurenic acid	Host, Microbiota	217 μM	Not available	66 μM	(23, 42, 43)
LPA	Host, Microbiota	Not available	Not available	Not available	(20, 24)
CXCL17	Host	Not available	Not available	Not available	(25)
DHICA	Host	22 μM	Not available	Not available	(44)
Reverse T3	Host, Microbiota	100 μM	Not available	Not available	(44, 45)
cGMP	Host	131 μM	Not available	Not available	(46)
**Synthetic Agonists**		**EC50 (human)**	**EC50 (mouse)**	**EC50** **(rat)**	**References**
Zaprinast		2-8 μM	1 μM	100 nM	(19, 42)
Lodoxamide		4 nM	Not available	13 nM	(47)
Pamoic acid		30-50 nM	No response	>100 μM	(17, 42)
Amlexanox		Not available	Not assessed	Not available	(48, 49)
Bufrolin		13 nM	Not available	10 nM	(47)
Compound 1		26 nM	17 μM	8 μM	(50)
Cromolyn disodium		Not available	Not available	Not available	(42, 49)
6-bromo-8-(4-methoxybenzamido)-4-oxo-4H-chromene-2-carboxylic acid		Not available	Not assessed	Not available	(51)
**Antagonists**		**IC50** **(human)**	**IC50** **(mouse)**	**IC50** **(rat)**	**References**
ML-145		~25 nM	No response	No response	(42)
CID2745687		~200 nM	No response	No response	(17, 42)
ML-194		~200 nM	Not assessed	Not assessed	(52)

### Synthetic Agonists

#### Zaprinast

Although there has been significant progress in identifying the putative endogenous activators of GPR35, usage of synthetic agonists also proved useful in revealing the molecular pathways involved in GPR35 activation. Of these synthetic agonist, Zaprinast a cyclic guanosine monophosphate-specific phosphodiesterase inhibitor, is one of the most widely known agonists of GPR35 (19). In contrast to the other compounds activating GPR35, zaprinast shows similar potency for human, rat, and mouse GPR35, and therefore serves as an optimal reference in studies (34).

#### Lodoxamide

Lodoxamide, an anti-inflammatory mast cell stabilizer, is another synthetic agonist for GPR35. However, although potency of lodoxamide is high for human and rat GPR35, it is 100-fold lower for the mouse orthologue (47, 53). Nevertheless, lodoxamide has shown to be protective in hepatic fibrosis model in mice which was reversed by a GPR35 antagonist (53). However, due to the concerns over lodoxamide’s low potency for mouse GPR35, it remains controversial whether this effect is caused by direct activity of GPR35 or whether there are other molecular pathways involved.

Other synthetic agonists of GPR35 include amlexanox, bufrolin, compound 1, pamoic acid, cromolyn disodium and, 6-bromo-8-(4-methoxybenzamido)-4-oxo-4H-chromene-2-carboxylic acid (**Table 1**). However, most of these compounds either were only tested on human GPR35 expressing cells but not rodent orthologues or have low potencies in the latter species, limiting their usage in rodent studies (18, 48–51).

### Antagonists

Receptor antagonists, by definition, are ligands that lead to inhibition of the activity of a receptor. They are crucial for functional studies as well as for investigating protein-protein interactions of receptors. ML-145 is one of the described GPR35 antagonists. It has a higher binding capacity for human GPR35 than mouse and rat orthologues and therefore have been rather used for *in vitro* settings rather than rodent models (42). Similar results were obtained for CID2745687 where both antagonists led to effective inhibition of agonist activities of zaprinast and cromolyn disodium for human GPR35. On the other hand, in this study, neither ML-145 nor CID2745687 have shown antagonism for rodent orthologues of GPR35. Interestingly, Zhao et al. found that CID2745687 is able to inhibit pamoic acid induced GPR35 activation (17). In addition, CID2745687 antagonized kynurenic acid in mouse astrocytes and interfered with pamoic acid-mediated wound repair in young adult mouse colon epithelium cells (54, 55). Lastly, ML-194 has also been introduced as a GPR35 antagonist which remains to be confirmed (52).

## Risk Variants of GPR35

Genome-wide association studies (GWAS) identified 70 SNPs in the coding region of GPR35 gene and the intergenic regions surrounding it. Six of these SNPs have been associated with increased risk of immune-related diseases (**Table 2**) (26, 69). These diseases include IBD, both ulcerative colitis and Crohn’s disease, as well as primary sclerosing cholangitis (PSC) (27, 28). Among the IBD-associated SNPs, the rs3749171 variant that causes a threonine to methionine transition in the transmembrane III, T108M, is the most extensively studied. Schneditz and colleagues showed that this variant is hypermorphic leading to hyperactivation of GPR35 (36). In this study, GPR35 T108M resulted in increased proliferation and metabolism in BMDMs and intestinal epithelial cells. In addition, another study from the same group demonstrated that macrophages carrying the T108M have enhanced VEGF and CXCL-8 production as opposed to the reduced levels in GPR35-deficient cells further reinforcing the hyperactivity caused by the variant (70). In addition to these studies, we reported that T108M variant correlated with better clinical outcome in response to TNF blockers IBD patients (20). Notably, in our study, GPR35 signaling facilitated macrophage TNF responses. It is possible that the aberrant TNF resulting from the hypermorphic T108M variant contributes to IBD pathogenesis and therefore leads to a better response to anti-TNF treatment. However, this potential mechanism remains a speculation and need to be studied more extensively. The T108M variant did not affect the agonist potencies for GPR35 profoundly, therefore, the mechanism by which the GPR35 T108M drives the hyperactivation remains unknown (47). The second IBD-associated SNP in GPR35 is rs4676410 which is an upstream intron variant causing a cytosine to thymine substitution. Although rs4676410 has a higher prevalence than T108M (**Table 3**), its function remains to be studied to date. Nevertheless, it is known that it does not cause a change in GPR35 tissue expression in IBD patients (27).

**Table 2:** Risk variants of GPR35 associated with diseases.

Variant ID	Variant type	Position (ch2):	Substitution	MAF	Linked Diseases	References
rs4676410	Intron	240624322	G>A	0.272	Ankylosing spondylitis, IBD, PSC, systemic lupus erythematosus	(27, 28, 56–59)
rs3749171	Missense	240630275	C>T (T108M)	0.151	CD, UC	(59–62)
rs3749172	Missense	240630832	C>A (S294R)	0.484	CD, coronary artery calcification	(63, 64) (65)
rs4676408	Intergenic	240634984	A>G	0.4	UC, CD	(66)
rs4676406	Intergenic	240639691	T>G	0.374	Ankylosing spondylitis, psoriasis, PSC	(60, 67)
rs34236350	5’ UTR	240628909	C>T	0.261	Ankylosing spondylitis, psoriasis	(68)

MAF, Minor Allele Frequency; UTR, Untranslated region.

**Table 3:** Haplotype frequencies of IBD-associated GPR35 variants across all populations analyzed by LDHap (ldlink.nci.nih.gov/?tab=ldhap).

RS Number	Position (GRCh37)	Allele Frequencies	Haplotypes		
rs4676410	chr2:241563739	G=0.728, A=0.272	G	A	A
rs3749171	chr2:241569692	C=0.849, T=0.151	C	T	C
		**Haplotype Count**	**3636**	**744**	**617**
		**Haplotype Frequency**	**0.726**	**0.1486**	**0.1232**

## GPR35 in Intestinal Health and Disease

IBD is a collective term to define chronic inflammatory conditions in the gastrointestinal tract including CD and UC. Patients with IBD experience a severely compromised quality of life with symptoms diarrhea, weight loss, abdominal pain, and are more susceptible to colorectal cancer (71). Current IBD treatment options pose a significant burden on the health system due to high costs and resource exhaustion. There is an increasing prevalence for IBD globally particularly in western countries due to the urbanization of life style (72).

**Figure 1:**
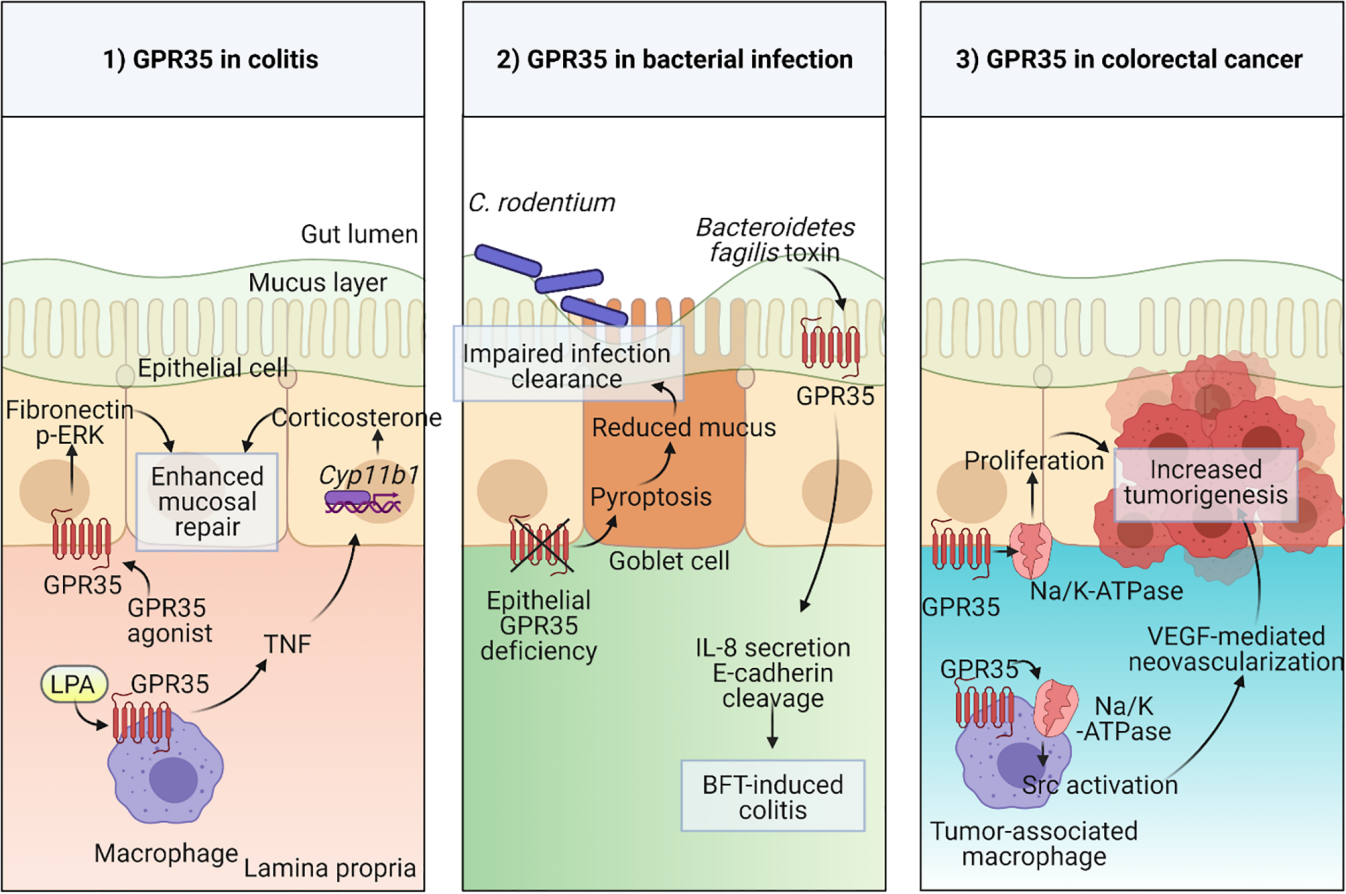
Physiological functions of GPR35 in the intestine 1) Macrophage expressed GPR35 induces TNF accompanied by transcription of *Cyp11b1* encoding for steroidogenic enzyme 11β –hydroxylase in epithelial cells during colitis. In a second mechanism, epithelial GPR35 promotes mucosal repair through ERK and fibronectin-mediated proliferation. 2) Disruption of GPR35 signaling in epithelial cells leads to pyroptosis in goblet cells resulting in reduced mucus production and therefore an impaired mucosal barrier integrity which leads to increased susceptibility to *C. rodentium* infection. GPR35 serves as a sensor in *Bacteroidetes fragilis* toxin (BFT)-induced colitis. 3) GPR35 interaction with Na/K-ATPase pump induces Src activation in both epithelial cells and tumor-associated macrophages (TAMs) in colorectal cancer model *via* increased proliferation in epithelial cells and neovascularization in response to TAM-derived VEGF. Created with BioRender.com.

The abundant expression of GPR35 in the intestine and its IBD-associated variants give hints towards an important physiological function for GPR35 in the gut. To explore this possibility, researchers adopted several experimental approaches including *in vitro* culture systems and disease models using rodents. For instance, in a wound healing model, GPR35 agonists, YE120, zaprinast and pamoic acid, promoted wound repair in young adult mouse colon epithelium cells *via* increased migration by fibronectin expression and ERK phosphorylation (55). Furthermore, several lines of evidence from multiple studies have shown that GPR35 is protective in dextran sodium sulfate (DSS) induced colitis mouse model (20, 55, 73). In this model, DSS causes cell death epithelial cells causing a breach in the barrier integrity and therefore dissemination of microbial products which leads to acute inflammation (74). Similar to the wound healing model, GPR35 agonists stimulated fibronectin and ERK activation in the colonic epithelium and therefore facilitated mucosal repair in the DSS model (**Figure 1**) (55). In subsequent studies, including ours, GPR35-deficiency led to exacerbated DSS colitis in mice as measured by clinical signs such as weight loss (20, 73). Nonetheless, these studies lack the genetic models required to dissect the cell specific effects of GPR35.

Our studies described the GPR35 expression across cell types in the intestine where the expression was prominent in epithelial cells as well as macrophages and dendritic cells. The macrophages in the intestine are continuously generated from the extravasating Ly6C^high^ blood monocytes through their intermediates (75). They are vital for the maintenance of tolerance at the steady state and search the lumen for antigens by extending their dendrites between epithelial cells (76–78). On the other hand, they can also contribute to inflammatory conditions by producing pro-inflammatory cytokines including TNF (79). Interestingly, in the DSS colitis model, macrophage-specific GPR35 deletion led to elevated inflammation which was accompanied by reduced TNF responses in macrophages as well as intestinal corticosterone production (**Figure 1**) (20). TNF is widely known for its pro-inflammatory effects and its clinically routine use as a target in IBD treatment strategies (80, 81). Nevertheless, it can also play an anti-inflammatory role through corticosterone production in intestinal epithelial cells which is in line with our experimental findings (82, 83). In a more recent study, Pagano et al. revealed that macrophage-expressed GPR35 promotes neoangiogenesis *via* VEGF production and therefore tumor growth in colitis associated and spontaneous models of colon cancer in mice (70). Since neovascularization is not only involved in cancer but also in IBD, this study potentially highlights yet another mechanism by which the GPR35 signaling in macrophages plays a role in IBD pathogenesis (84).

The physiological relevance of epithelial GPR35 in the gut has also been the focus of several studies, since GPR35 is strongly expressed in intestinal epithelial cells (20). As an example, a study from our group explored the role for GPR35 in goblet cell function at the steady state and during *Citrobacter rodentium* infection (85). Deficiency of GPR35 in epithelial cells leads to a decrease in goblet cell numbers and Muc2 expression due to enhanced pyroptosis of goblet cells. Pyroptosis a cell death mechanism where the active caspase-1 and caspase-11 cleave gasdermin D causing pore formation in the cell membrane (86). By inducing pyroptosis, epithelial GPR35 deficiency led to compromised epithelial barrier and therefore susceptibility to *Citrobacter rodentium* infection (**Figure 1**). On the other hand, a recent study has shown that epithelial GPR35 is crucial in sensing the enterotoxigenic *Bacteroides fragilis* toxin (BFT) and initiates an immune response against it (**Figure 1**) (87). Therefore, as opposed to the *Citrobacter rodentium* infection model, epithelial GPR35 deficiency led to protection from BFT-induced colitis. Together, these two studies emphasize that the role of GPR35 signaling in epithelial cells is context-dependent in bacterial infections. Lastly, Schneditz and colleagues examined the physiological relevance of epithelial GPR35 signaling in spontaneous colon cancer model in mice (**Figure 1**) (36). Deletion of epithelial GPR35 led to reduced proliferation and tumor burden in this model, showing that in GPR35 contributes to cell turn over in epithelial cells during tumorigenesis.

## Conclusion

GPR35 is an emerging GPCR in the intestinal health field with potential implications in IBD as well as colorectal cancer. GPR35 might prove useful as an intermediate player and a messenger between the microbiota and host *via* metabolite-sensing. However, despite the experimental evidence pointing towards potential endogenous ligand candidates for GPR35, the biochemistry of GPR35 is not fully understood. The challenges in the context of GPR35 pharmacology include orthologue selectivity, off-target effects, and lack of knowledge on interactions that are based on direct binding. To overcome these, better pharmacological assays need to be carried out to get a broader overview of the GPR35 signaling.

In this review, we summarized the recent advances in understanding the physiological functions of GPR35 and its IBD-associated risk variant T108M particularly in intestinal macrophages and epithelial cells. However, before GPR35 can be considered as a pharmaceutical target for the treatment of intestinal diseases, more studies are needed to elucidate the mechanisms governed by GPR35 signaling.

## Author Contributions

JN and BK conceived the content and structure of the manuscript. BK drafted the figures and JN, BK, and HM wrote the text. All authors contributed to the article and revised the final version.

## Funding 

The SNSF grant 310030_175548 to JN supported this manuscript’s writing.

## Conflict of Interest

The authors declare that the research was conducted in the absence of any commercial or financial relationships that could be construed as a potential conflict of interest.

## Publisher’s Note

All claims expressed in this article are solely those of the authors and do not necessarily represent those of their affiliated organizations, or those of the publisher, the editors and the reviewers. Any product that may be evaluated in this article, or claim that may be made by its manufacturer, is not guaranteed or endorsed by the publisher.
